# Impact of bronchopulmonary dysplasia on brain and retina

**DOI:** 10.1242/bio.017665

**Published:** 2016-03-17

**Authors:** Annie Wing Hoi Poon, Emilie Xiao Hang Ma, Arul Vadivel, Suna Jung, Zehra Khoja, Laurel Stephens, Bernard Thébaud, Pia Wintermark

**Affiliations:** 1 Division of Newborn Medicine, Department of Pediatrics, McGill University, Montreal, Quebec H4A 3J1, Canada; 2 Ottawa Hospital Research Institute, Regenerative Medicine Program, Department of Pediatrics, Children's Hospital of Eastern Ontario, University of Ottawa, Ottawa, Ontario K1H 8L6, Canada

**Keywords:** Brain, Bronchopulmonary dysplasia, Chronic lung disease, Encephalopathy of prematurity, Hyperoxia, Lung, Newborn, Retina, Retinopathy of prematurity

## Abstract

Many premature newborns develop bronchopulmonary dysplasia (BPD), a chronic lung disease resulting from prolonged mechanical ventilation and hyperoxia. BPD survivors typically suffer long-term injuries not only to the lungs, but also to the brain and retina. However, currently it is not clear whether the brain and retinal injuries in these newborns are related only to their prematurity, or also to BPD. We investigated whether the hyperoxia known to cause histologic changes in the lungs similar to BPD in an animal model also causes brain and retinal injuries. Sprague Dawley rat pups were exposed to hyperoxia (95% O_2_, ‘BPD’ group) or room air (21% O_2_, ‘control’ group) from postnatal day 4–14 (P4–14); the rat pups were housed in room air between P14 and P28. At P28, they were sacrificed, and their lungs, brain, and eyes were extracted. Hematoxylin and eosin staining was performed on lung and brain sections; retinas were stained with Toluidine Blue. Hyperoxia exposure resulted in an increased mean linear intercept in the lungs (*P*<0.0001). This increase was associated with a decrease in some brain structures [especially the whole-brain surface (*P*=0.02)], as well as a decrease in the thickness of the retinal layers [especially the total retina (*P*=0.0008)], compared to the room air control group. In addition, a significant negative relationship was observed between the lung structures and the brain (r=−0.49, *P*=0.02) and retina (r=−0.70, *P*=0.0008) structures. In conclusion, hyperoxia exposure impaired lung, brain, and retina structures. More severe lung injuries correlated with more severe brain and retinal injuries. This result suggests that the same animal model of chronic neonatal hyperoxia can be used to simultaneously study lung, brain and retinal injuries related to hyperoxia.

## Abstract (precis)

**Summary:** Our results suggest that the same animal model of chronic neonatal hyperoxia can be used to simultaneously study lung, brain and retinal injuries related to hyperoxia.

## INTRODUCTION

Prematurely born newborns represent 5–12% of all births (Beck et al., 2010; Landry et al., 2011), and they are at a high risk of long-term developmental complications. Contemporary advances in maternal and neonatal care have enabled the survival of very preterm infants (i.e. 24–32 weeks gestation) who are vulnerable during the first months of their life to lung, brain, and retinal impairments, since these organs are still maturating during those months. Encephalopathy of prematurity (EOP) (Volpe, 2009) and retinopathy of prematurity (ROP) (Cavallaro et al., 2014; Rivera et al., 2011; Sapieha et al., 2010) are the two main contributors to long-term neurodevelopmental sequelae, such as cerebral palsy, intellectual disability, learning difficulties, blindness, and other sensory and cognitive deficits.

In addition, very preterm infants often require chronic mechanical ventilation for a prolonged period of time, as well as exposure to high and variable oxygen levels (Weinberger et al., 2002). Prolonged mechanical ventilation and exposure to high levels of oxygen can cause inflammation and oxidative stress in the lungs and lead to an arrest of the lungs' alveolar and microvascular growth, resulting in a chronic respiratory disease known as bronchopulmonary dysplasia (BPD) (Baker and Abman, 2015; Baraldi and Filippone, 2007). BPD survivors not only suffer from long-term injuries to the lungs and respiratory complications later in life, but they also present an additional risk of developing brain and retinal injuries (Brown et al., 1990; Natarajan et al., 2012; Schmidt et al., 2003; Trittmann et al., 2013). However, currently, it is not clear whether the brain and retinal injuries in these newborns are related only to their prematurity, or also to BPD.

In this study, we used a rodent model of chronic neonatal hyperoxia that mimics the histological changes in the lungs observed in BPD (O'Reilly and Thébaud, 2014) to explore the effects of hyperoxia on the brain and retina. We hypothesized that the hyperoxia known to cause changes mimicking BPD in this animal model also causes brain and retinal injuries similar to those observed in premature human newborns.

## RESULTS

### Hyperoxia impaired lung alveolarization

**Fig. 1.**
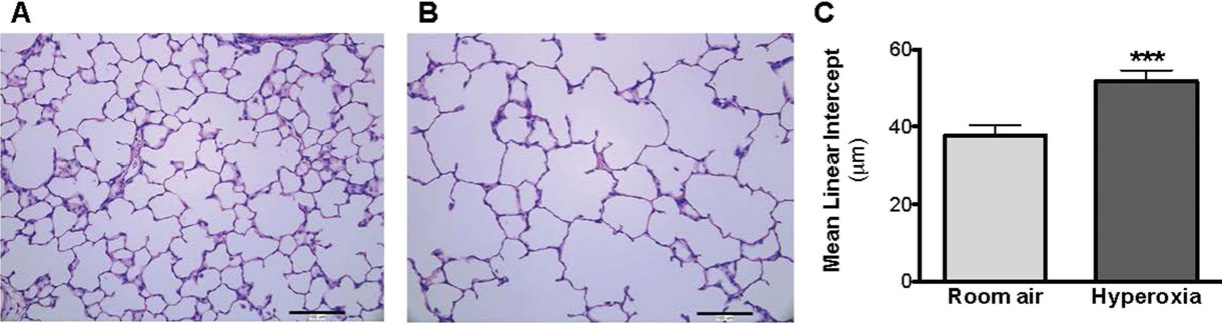
**Hematoxylin and eosin-stained lung sections of P28 old rats exposed to room air (‘control’ group) or hyperoxia (‘BPD’ group) from P4 to P14.** (A,B) Newborn rats were housed in 95% O_2_ from P4 to P14 and studied in comparison with control rats raised in room air. The exposure of rat pups to hyperoxia (B) during the alveolar stage of lung development results in arrested alveolar growth characterized by larger and fewer alveolar structures compared to (A) room air rat pups, as shown in hematoxylin and eosin-stained representative lung slides. (C) The mean linear intercept was measured to quantify alveolar structures. Data represented as mean±s.d.; ***P<0.001, comparison versus room air control group.

**Fig. 2.**
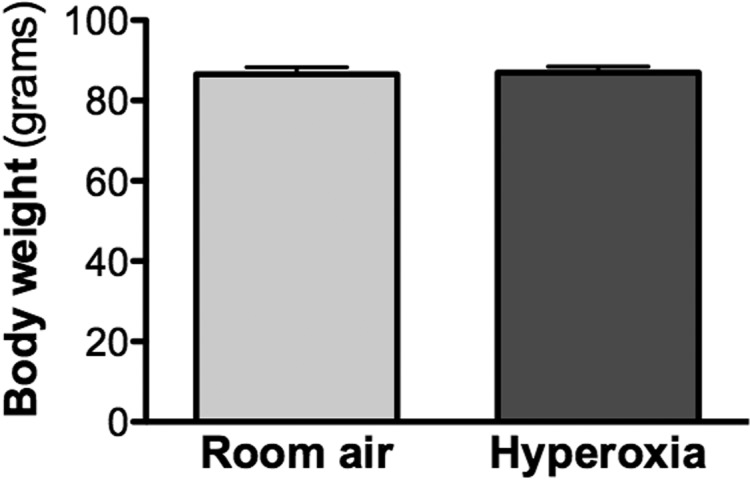
**Body weight of P28 old rats exposed to room air (‘control’ group) or hyperoxia (‘BPD’ group) from P4 to P14.** A comparison of the body weights of rats housed in 95% O_2_ from P4 to P14 and weighed at P28 found no significant difference in comparison with control rats raised in room air. Data represented as mean±s.d.

The lungs of the rat pups exposed to hyperoxia from postnatal day (P)4 to P14 developed a histological pattern reminiscent of human BPD, which was characterized by fewer and enlarged alveolar structures. The mean linear intercept was increased significantly at P28 in the hyperoxia group (51.79±2.79 μm, *P*<0.0001), compared to the room air control group (37.72±2.61 μm; Fig. 1). Of note, the body weight was not different at P28 between the hyperoxia group (86.98±4.77 g) and the room air control group (86.57±5.38 g; *P*=0.79; Fig. 2).

### Hyperoxia induced a significant decrease in the size of several brain structures, correlating with the lung alveolar structure impairment

**Fig. 3.**
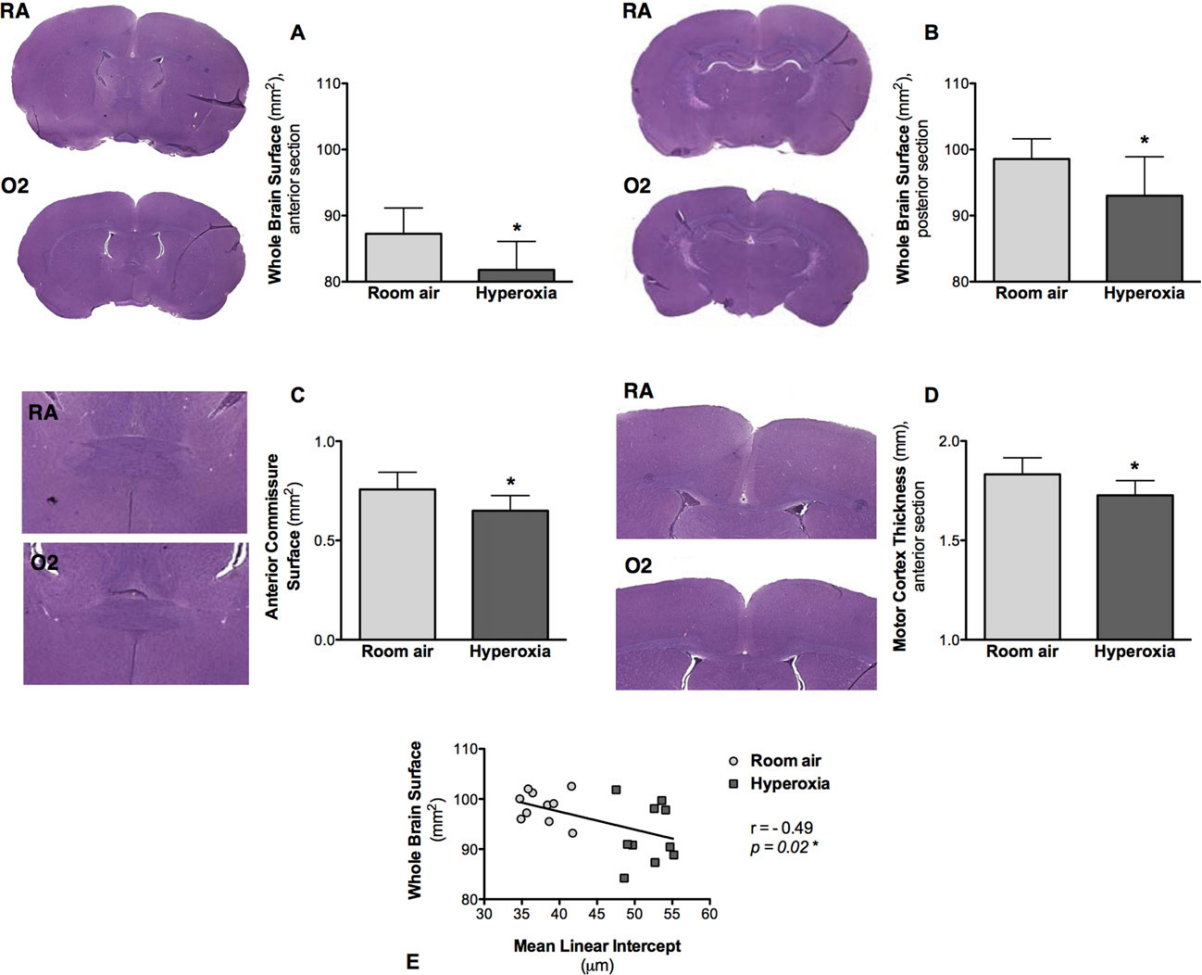
**Hematoxylin and eosin-stained coronal brain sections of P28 old rats exposed to room air (‘control’ group) or hyperoxia (‘BPD’ group) from P4 to P14.** (A-D) Representative coronal brain sections and measurements of different brain structures. (A) Whole-brain surface measured on the anterior sections. (B) Whole-brain surface measured on the posterior sections. (C) Surface of the anterior commissure measured on the anterior sections. (D) Thickness of the cortex measured on the anterior sections from the horn of the corpus callosum to the outer limit of the brain, parallel to the midline. Data represented as mean±s.d.; *P<0.05, in the oxygen-exposed (O_2_) rat pups, compared to the room air-exposed (RA) rat pups. (E) Comparison between the whole-brain surface measured on the posterior sections (reflecting brain structure) and the mean linear intercept (reflecting lung alveolar structure), showing a negative correlation between both structures (r=−0.49, *P*=0.02).

The hyperoxia-exposed rat pups displayed a significant decrease in the size of several brain structures (Table 1). Specifically, hyperoxia caused a significant reduction in the size of the whole brain surface in the anterior and posterior sections in the exposed rat pups, compared to the room air control group: respectively, 81.79±4.30 mm^2^ vs 87.24±3.90 mm^2^ (*P*=0.02) in the anterior sections, and 93.00±5.90 mm^2^ vs 98.56±3.05 mm^2^ (*P*=0.04) in the posterior sections (Fig. 3A, Fig. 2B). The surface of the anterior commissure also decreased in the rat pups exposed to hyperoxia (0.65±0.07 mm^2^, *P*=0.01), compared to the room air control group (0.76±0.09 mm^2^; Fig. 3C). The thickness of the cortex decreased in the hyperoxic animals: respectively, 1.73±0.07 mm vs 1.83±0.08 mm (*P*=0.03) in the anterior sections and 1.54±0.04 mm vs 1.62±0.08 mm (*P*=0.07) in the posterior sections (Fig. 3D). The width of the corpus callosum tended to be smaller in the hyperoxia-exposed rat pups, but the difference was not significant compared to the room air control group: respectively, 0.25±0.03 mm vs 0.29±0.02 mm (*P*=0.06) in the anterior sections, and 0.30±0.01 mm^2^ vs 0.32±0.01 mm (*P*=0.26) in the posterior sections. There was no difference between the groups with respect to the surface of the hippocampus.

**Table 1.**
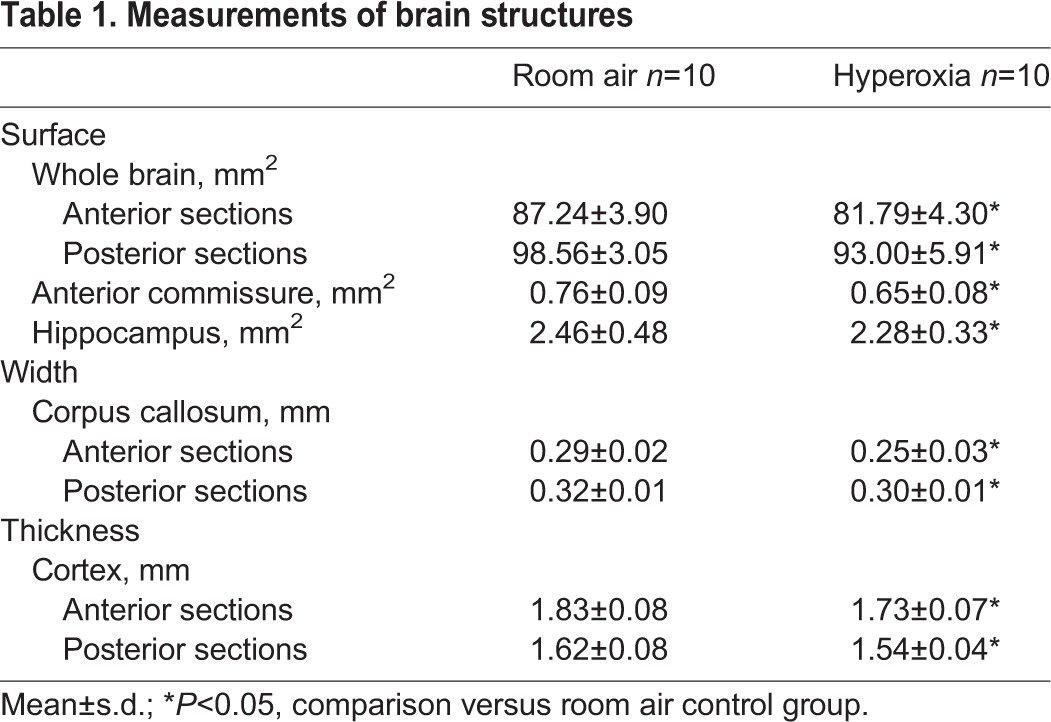
**Measurements of brain structures**

When looking at the relationship between brain injury and lung injury, statistical analysis using Spearman's rank correlations showed a moderate negative correlation between the whole-brain surface and the mean linear intercept (r=−0.49, *P*=0.02; Fig. 3E).

### Hyperoxia led to a retinopathy, correlating with the lung alveolar structure impairment

**Fig. 4.**
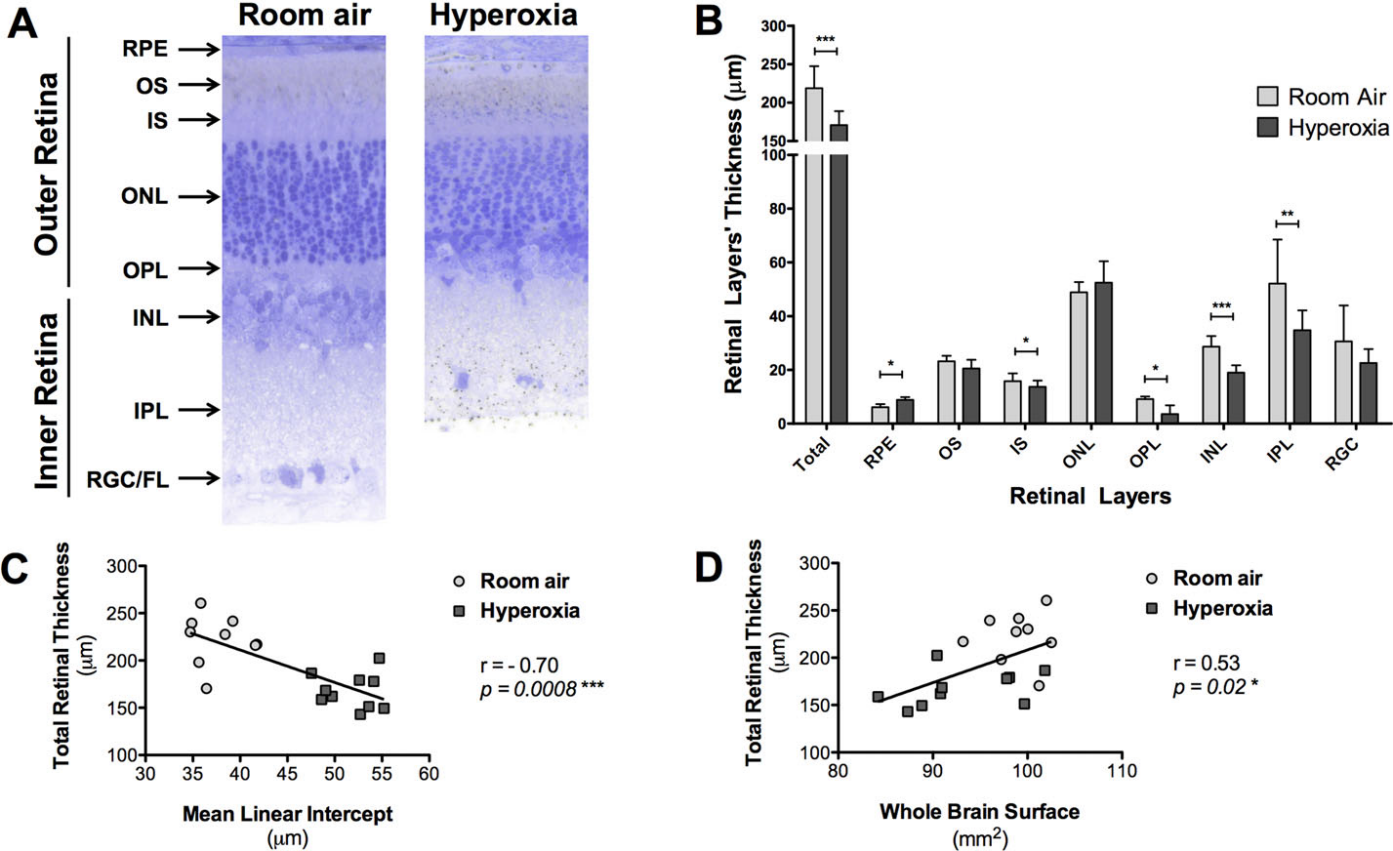
**Toluidine Blue-stained retinal cross sections from the eyes of P28 old rats exposed to room air (‘control’ group) or hyperoxia (‘BPD’ group) from P4 to P14.** (A) Representative retinal cross sections, images were taken at approximately 1000 µm from the optic nerve head in the inferior retina. (B) Thickness of the retinal layers measured at 1000 µm from the optic nerve head. RPE, retinal pigment epithelium; OS, outer segment; IS, inner segment; ONL, outer nuclear layer; OPL, outer plexiform layer; INL, inner nuclear layer; IPL, inner plexiform layer; RGC/FL, retinal ganglion cell/fiber layer. Data represented as mean±s.d.; *P<0.05, **P<0.01, ***P<0.001 in the hyperoxia-exposed rat pups compared to the room air-exposed rat pups*.* (C) Comparison between the average total retinal thickness measured (reflecting retinal structure) and the mean linear intercept (reflecting lung alveolar structure), showing a negative correlation between both structures (r=−0.58, *P*=0.009). (D) Comparison between the average total retinal thickness measured (reflecting retinal structure) and the whole-brain surface measured on the posterior sections (reflecting brain structure), showing a positive correlation between both structures (r=0.55, *P*=0.01).

The hyperoxia induced changes in the retinal structure in the exposed rat pups (Table 2; Fig. 4). The total retinal thickness was significantly decreased in the hyperoxic retinas (170.67±17.46 µm, *P*=0.0008), compared to the control retinas exposed only to room air (222.32±26.39 µm; Fig. 4A). An analysis of the thicknesses of the individual retinal layers at 1000 µm also revealed significant differences between the groups. The rat pups exposed to hyperoxia displayed a significant decrease in thicknesses at 1000 µm of the following layers as compared to the room air control group: the inner segment (IS) (11.98±3.62 µm vs 16.41±3.97 μm, *P*=0.04), the outer plexiform layer (OPL) (4.60±3.68 µm vs 9.17±1.42 µm, *P*=0.01), the inner nuclear layer (INL) (18.32±3.47 µm vs 30.12±4.90 µm, *P*<0.0001), and the inner plexiform layer (IPL) (34.00±8.40 µm vs 54.06±14.45 µm, *P*=0.009; Fig. 4B). The retinal pigmental epithelium layer (RPE) was significantly thicker in the hyperoxic animals as compared to the room air-exposed pups (8.87±1.34 µm vs 6.55±1.28 µm, *P*=0.01). In contrast, the thicknesses of the outer segment (OS), the inner segment (IS), the outer nuclear layer (ONL), and the retinal ganglion cell/fiber layer (RGC/FL) were not significantly different between the two groups.

**Table 2.**
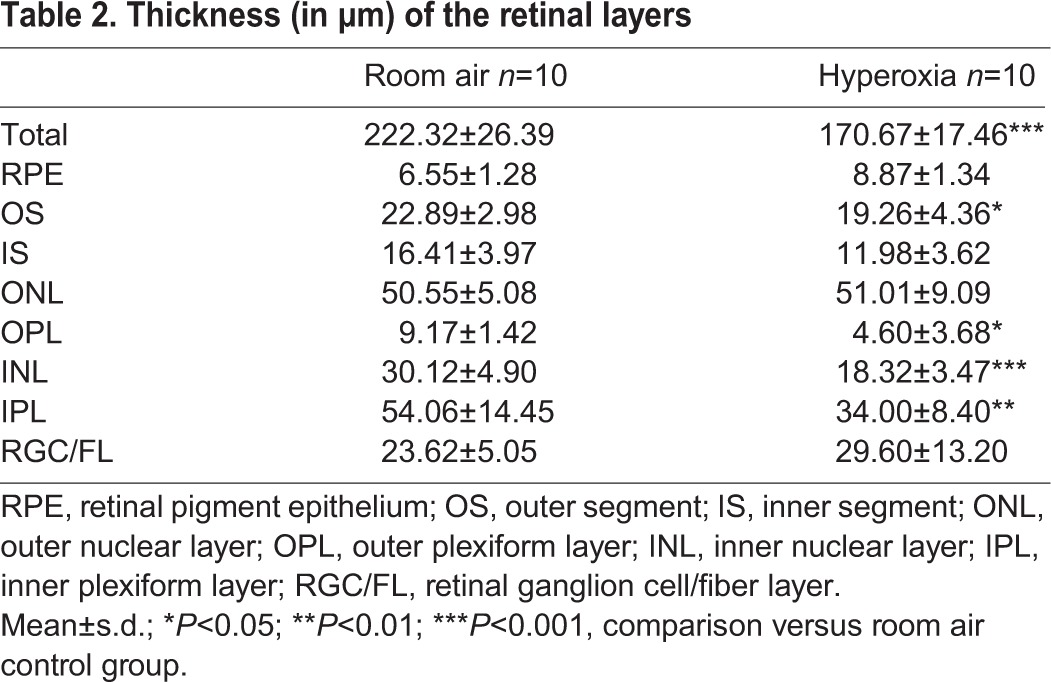
**Thickness (in μm) of the retinal layers**

When looking at the relationship between retinal injury and lung injury, statistical analysis using Spearman correlations showed a strong negative correlation between the total retinal thickness and the mean linear intercept (r=−0.70, *P*=0.0008; Fig. 4). When looking at the relationship between retinal injury and brain injury, a moderate positive correlation was observed between the total retinal thickness and the whole-brain surface (r=0.53, *P*=0.02; Fig. 4).

**Fig. 5.**
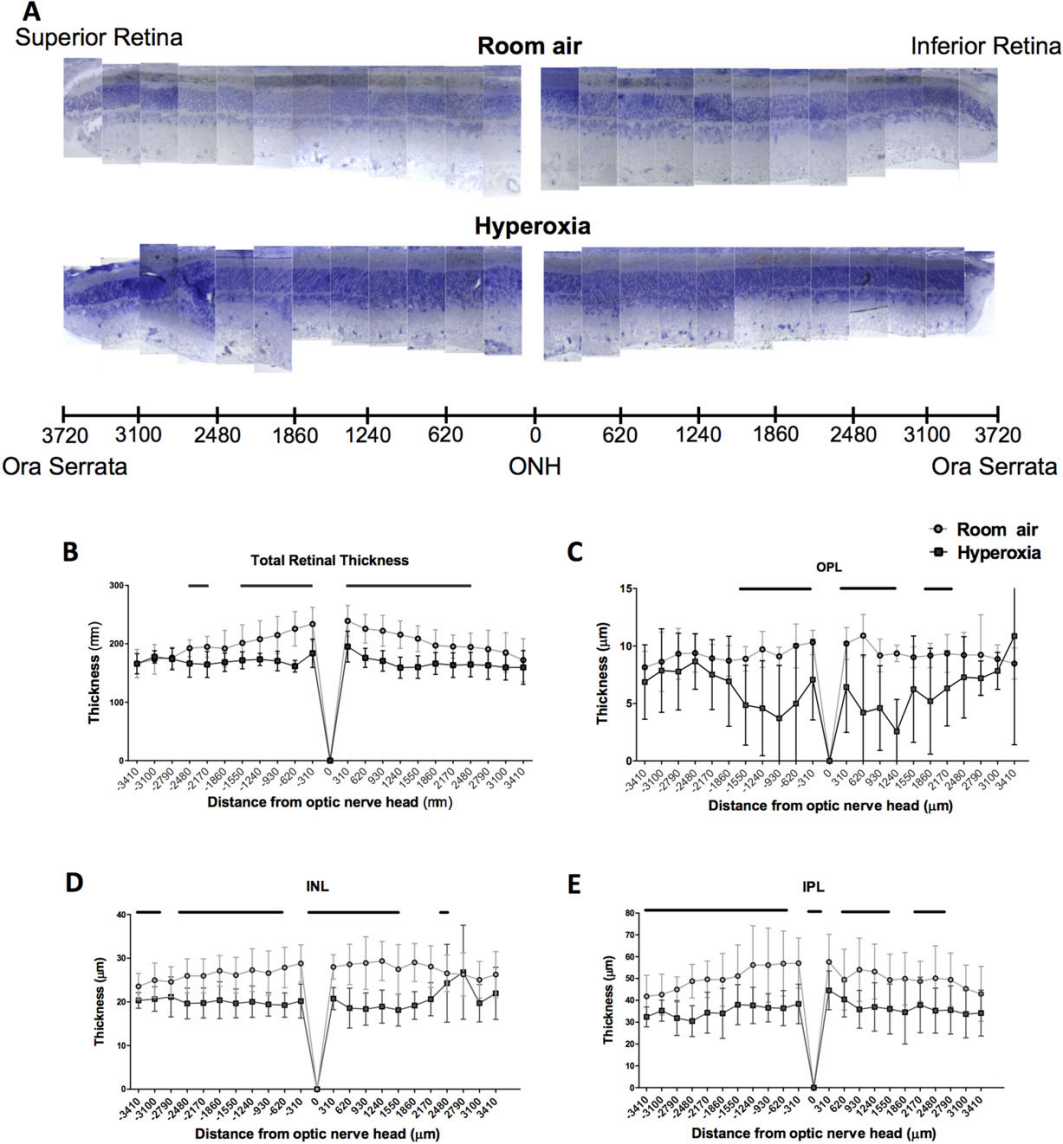
**Topographic distribution of the retinal injury.** (A) Representative pan-retinal view of P28 old rats exposed to room-air (‘control’ group) or hyperoxia (‘BPD’ group). (B-E) Spider graphs depicting the variation in (B) total retina, (C) OPL, (D) INL, and (E) IPL thicknesses with eccentricity. ONH, optic nerve head; OPL, outer plexiform layer; INL, inner nuclear layer; IPL, inner plexiform layer. Data represented as mean±s.d. BPD groups compared to the control group: significance (*P*<0.05) represented by black lines at the top of each graph. Retinal section thicknesses differed significantly between the control group and the BPD group according to the distance from the optic nerve head.

The distribution of the retinal injury was heterogeneous along the superior-inferior axis of the retina: i.e. the damage was more pronounced at the center compared to the periphery of the retina (Fig. 5). In the hyperoxic retinas compared to the normoxic retinas, the thicknesses of the IS, OPL, INL, and IPL were significantly reduced in the central part of the retina (within 1240 µm of the optic nerve head), but not at the periphery of the retina (past 1550 µm from the optic nerve head).

## DISCUSSION

In the present study, we have demonstrated that the hyperoxia known to cause lung structural anomalies reminiscent of human BPD in premature rat pups also is one of the responsible mechanisms causing brain and retinal injuries in these animals. In the present study, the hyperoxia exposure of the rat pups resulted in an increased mean linear intercept in the lungs, in smaller brain structures, and in a decrease in the thickness of the retinal layers, compared to the room air control group. A difference in body weight did not seem to explain the smaller structures in the lungs, brain and retina (Balasubramaniam et al., 2010; Rieger-Fackeldey et al., 2014). Together, these results suggest that the same animal model of BPD can be used to study lung, brain, and retinal injuries.

In our experiments, exposure to hyperoxia led to smaller brain structures as observed by the histology. In particular, the whole-brain surface, the surface of the anterior commissure, as well as the cortical thickness were significantly decreased in the hyperoxia-exposed rat pups compared to the room air control group. Our findings in this chronic neonatal hyperoxia model (continuous exposure to 95% oxygen for 10 days) are consistent with previous studies that specifically examined the damaging effect of hyperoxia in the brain by using different premature rodent models (exposure to 80% oxygen for a shorter period of time ranging from 2 h to 6 days) (Felderhoff-Mueser et al., 2004; Gerstner et al., 2008; Kurul et al., 2009; Pham et al., 2014; Ritter et al., 2013; Sirinyan et al., 2006; Vottier et al., 2011; Yiş et al., 2008). Yiş et al. (2008) exposed Wistar rat pups to 80% oxygen from birth to P5 and found a decrease in brain weight in the exposed animals. Sirinyan et al. (2006) exposed Sprague Dawley rat pups to 80% oxygen from birth to P6 and also found a decrease in brain weight. In humans, encephalopathy of prematurity (EOP) (Volpe, 2009) is typically associated with a decreased volume of brain structures – such as the white matter, thalamus, basal ganglia, cerebral cortex, and cerebellum – at a term-equivalent age, and also later in childhood, adolescence, and adulthood (Bjuland et al., 2014; Martinussen et al., 2009; Woodward et al., 2006). As the brain develops, it undergoes a period of rapid growth during which it is particularly susceptible to environmental factors (Ikonomidou and Kaindl, 2011). In rats, this rapid brain growth period occurs within the first three postnatal weeks (Ikonomidou and Kaindl, 2011). Thus, exposing rat pups to 95% oxygen from P4 to P14 subjects their developing brain to increased oxidative stress when it is the most vulnerable and its antioxidant mechanisms are still immature.

In our experiments, the anterior commissure and the cortex were the two most significantly affected brain structures, and the corpus callosum also tended to be smaller. Since the rapid increase in brain weight during the last semester is mainly due to glial cell multiplication (Ikonomidou and Kaindl, 2011) and this also is the time when the pre-oligeodendrocytes are very vulnerable (Back et al., 2001), it appears logical to suggest that insults to the premature brain during this period of growth will largely affect the brain white matter, which helps to explain why the surface of the anterior commissure was significantly decreased and also why the width of the corpus callosum tended to be smaller in the exposed animals in our experiments. Other researchers also have reported lower volumes of the hippocampal dentate gyrus and higher volumes of the subventricular zone in rats exposed to 60% or 95% oxygen from birth to P14 (Porzionato et al., 2015), as well as a decreased hippocampal and cerebellar size in mice exposed to 85% oxygen from P2 to P14 (Ramani et al., 2013) and impaired cortical matter between P3 and P7 (Felderhoff-Mueser et al., 2004). A hyperoxia-induced apoptosis appeared to be age-dependent (Ikonomidou and Kaindl, 2011). Thus, hyperoxia may affect different brain structures differently.

Only the sizes of the brain structures were examined in the present study; the underlying cellular and molecular mechanisms that could explain how hyperoxia led to smaller brain structures were not studied as already described in several other studies studying specifically the premature brain. Previous studies that specifically have examined the brain in rodent models exposed to hyperoxia described a decreased neuronal density (Felderhoff-Mueser et al., 2004; Yiş et al., 2008; Kurul et al., 2009), decreased microvascular density (Sirinyan et al., 2006), decreased microglial activation (Vottier et al., 2011; Pham et al., 2014), oligodendrocyte apoptosis (Gerstner et al., 2008), and myelination abnormalities (Ritter et al., 2013). Consequently, brain injury can occur through widespread apoptosis, which leads to degeneration of the neurons (Vottier et al., 2011; Gerstner et al., 2008) and impaired vasculogenesis that results in arrested neuron development (Sirinyan et al., 2006).

We also found that the continuous exposure of premature rat pups to 95% oxygen from P4 to P14 produced significant retinal structural damages. The total retinal thickness was decreased in the hyperoxia-exposed retina compared to the normoxic retina. In particular, the OPL, INL, and IPL were significantly thinner in the hyperoxic retinas compared to control retinas. The observed retinopathy shared similarities with the widely described retinopathy of prematurity (ROP) (Akula et al., 2010). Our findings were consistent with previous studies that specifically examined the damaging effect of hyperoxia in the retina by using different premature rodent models specifically targeted to the retina (Dembinska et al., 2001; Dorfman et al., 2010; Lachapelle et al., 1999). Compared to this chronic neonatal hyperoxia model, some popular ROP models have used an alternating oxygen exposure paradigm to create a hyperoxic/hypoxic retinal injury. One such typical model representative of ROP involves exposing rat pups to cycles of 24 h of hyperoxia using 50% oxygen followed by 24 h of hypoxia using 10% oxygen for 14 days (the 50%/10% alternating model) (Barnett et al., 2010). Using this 50%/10% alternating model, Akula et al. (2010) demonstrated that the OPL, INL, IPL, and RGC/FL layers were thinner at P21. In addition, by using another rodent paradigm including exposure to hyperoxia (80% oxygen) interrupted by three 0.5-h periods of normoxia (21% oxygen) per day for 14 days, Lachapelle et al. (1999), Dembinska et al. (2001), and Dorfman et al. (2010) found a significant thinning of the OPL. More specifically, Dembinska et al. (2001) found that the hyperoxic retinas exposed from P0 to P12–14 showed an 80% reduction in OPL thickness as compared to room air control retinas, whereas the pups exposed to hyperoxia only from P0 to P6–9 showed a 30% and 50% reduction of the OPL layer thickness, suggesting that P6–14 probably corresponds to the critical period of OPL growth, and the period in which hyperoxic exposure causes the most damage to the retina. In rat pups, the OS and OPL typically forms around P5 and the IPL around P12 (Weidman and Kuwabara, 1968), which further supports the hypothesis that P4–14 is the critical period of development for the retina, during which it is the most susceptible to hyperoxia, as shown in our study. In our experiments, the chronic neonatal hyperoxia model, which did not include any fixed exposure to room air (except very briefly every 48 h to change the dams) or hypoxia, still produced severe retinal cytoarchitectural damages, with a thinning of the whole retina, but more specifically the OPL, OS, INL, and IPL.

Only the retinal structure was examined in the present study; the underlying cellular and molecular mechanisms that could explain how hyperoxia leads to thinner retina were not studied as already described in several other studies studying specifically the premature retina. Previous studies that specifically examined the retina in rodent models have shown greater oxygen toxicity during the second week of life in rat pups (Dembinska et al., 2001) – retinal vascular abnormalities characterized by vaso-obliteration and abnormal vessel growth (Akula et al., 2010; Smith et al., 1994) and impaired rod photoreceptor and postreceptor responses (Akula et al., 2007; Lachapelle et al., 1999).

In addition, we showed that a significant correlation exists between the lungs, brain, and retinal injuries, which suggests that hyperoxia impaired the brain and retinal development with the same severity as the lungs. Interestingly, similar mechanisms of injury also have been described separately in these three organs in premature newborns. One of these similar mechanisms of injury that targets these three organs in premature newborns following exposure to hyperoxia is activated angiogenesis. In the lungs, vascular endothelial growth factor (VEGF) levels have been shown to be lower in infants with BPD compared to healthy infants without BPD (D'Angio and Maniscalco, 2002); in addition, treatment with VEGF promoted vessel growth and alveolar structure improvement in lungs of newborn rats subjected to hyperoxic conditions (Thébaud et al., 2005). In the brain, increased vascular endothelial growth factor (VEGF) expression has been described around the foci of the periventricular white matter injuries in the premature neonatal brain (Arai et al., 1998; Wintermark et al., 2015). With respect to the retina, VEGF is activated during the second phase of ROP, and the increased energy demands of the eye promote angiogenesis via the release of VEGF (Joyal et al., 2012).

Lung, brain, and retinal impairments are the three most common neonatal morbidities in very preterm infants, which strongly predict the risk of later death or neurosensory impairment (Schmidt et al., 2003). While previous animal studies have successfully demonstrated the damaging effects of hyperoxia separately on the lungs, brain, and retina, to our knowledge, the present study is the first demonstration that the same chronic neonatal hyperoxia animal model permits a simultaneous study of the oxygen-induced injuries of all three organs. We chose a well-established model of severe BPD (Berger and Bhandari, 2014; Hilgendorff et al., 2014; O'Reilly and Thébaud, 2014) to represent those preterm babies who will develop the most severe BPD forms and as such who will be the more at risk of developing brain and retinal injuries; these babies typically spend a few weeks on mechanical ventilation with fraction of inspired oxygen above 0.8 and receive other drugs (e.g. dexamethasone) that may further inhibit lung and brain growth. We also chose to compare the most commonly used structural measurements of each three organs, and not repeat the cellular and molecular assessments already demonstrated in each organ, so to study if there is a correlation between lung, brain and retinal injury and to better understand the impact of oxygen toxicity on lung, brain and retinal development. A single premature animal model that can be used to simultaneously study injuries in all three organs (lungs, brain, and retina) represents a unique opportunity for a more efficient development of therapeutic strategies for premature infants. Treatments developed to repair lung injuries may have similar effects in the brain and retina of premature newborns. For example, recently available treatments with stem cells have shown promising results for repairing premature lungs, both in terms of functional and structural improvement (Alphonse et al., 2014); however, the potential impact on the brain and the retina remains to be demonstrated. Exploring innovative therapeutic pathways that could simultaneously attenuate injuries in the lungs, brain, and retina of premature infants would provide a unique opportunity to contribute to the improvement of the future outcome of these newborns.

Studying three organs at the same time also may prove to be a strategy that will result in fewer animals being used for experiments, thus fulfilling one of the three Rs tenet that guides scientists in the ethical use of animals in science. Extracting the lungs, brain, and retina of the same animals instead of using separate animals for each organ will reduce the number of animals used for experiments.

In conclusion, we found that hyperoxia exposure not only impaired lung structures, but also those of the brain and retina. We found that an inverse relationship exists between measurements of lung structures and measurements of brain and retinal structures, which suggests that more severe lung injuries correlated with more severe brain and retinal injuries. These findings suggest that the same animal model of chronic neonatal hyperoxia exposure can be used to study simultaneously lung, brain, and retinal injuries related to hyperoxia. Further studies using this all-encompassing model should provide a better understanding of the development of brain and retinal injuries related to lung injuries in these newborns, and thus offer unique opportunities to develop innovative treatments targeting these three organs, and contribute to the improvement of the future outcome of these newborns.

## MATERIAL AND METHODS

### Animals

The experiments were approved by the local Animal Care Committee and conducted in accordance with the standard operating procedures for the use of animals in research as per the guidelines in the Canadian Council on Animal Care's Guide to the Care and Use of Experimental Animals, and in the Animals for Research Act. Adult female Sprague Dawley rats with their litters (Charles River Laboratories) were received in the animal facility, housed under a standard environment, and allowed food and water *ad libitum*. Rat pups remained with their mother until weaning at postnatal day 21 (P21). The rat pups were monitored daily by animal facility health care technicians, and additionally by the researchers during the course of the study.

### Chronic neonatal hyperoxia model

We chose a well-established model of severe BPD (Berger and Bhandari, 2014; Hilgendorff et al., 2014; O'Reilly and Thébaud, 2014) to represent those preterm babies who will develop the most severe BPD forms and as such who will be the more at risk of developing brain and retinal injuries. Newborn Sprague Dawley pups were randomly exposed to either room air (i.e. 21% oxygen; ‘control’ group) or hyperoxia (i.e. 95% oxygen; ‘BPD’ group) (*n*=10 animals in each group) from postnatal day 4 to 14 (P4–P14) in sealed Plexiglas chambers (OxyCycler, Biospherix, Lacona, NY) with continuous oxygen monitoring (Alphonse et al., 2014). Dams were switched every 48 h between the hyperoxic and normoxic chambers to avoid damage to their lungs and to ensure equal nutrition to each litter. At P14, all the rats were housed in room air until P28. At P28, all the rats were weighed and then sacrificed with an intraperitoneal injection of sodium pentobarbital (100 mg/kg); their lungs, brain, and eyes were extracted.

### Lungs

The lungs were fixed with a 10% neutral buffered formalin solution through the trachea under a constant pressure of 20 cm H_2_O. The trachea was then ligated, and the lungs were immersed in fixative overnight at 4°C. The lungs were processed and embedded in paraffin. Serial step sections, 4 μm in thickness, were taken along the longitudinal axis of the lobe. The fixed distance between the sections was calculated to enable a systematic sampling of 10 sections across the whole lobe (Alphonse et al., 2014; Thébaud et al., 2005). The lungs were stained with hematoxylin and eosin (H&E). The alveolar structures were quantified on a motorized microscope stage (Leica DM4000) using the mean linear intercept method (Quorum Technologies, Guelph, ON) (Alphonse et al., 2014; Thébaud et al., 2005; Tschanz, 2003). The mean linear intercept is one among several available lung measurements (i.e. lung function, secondary septal count, alveolar surface area and radial alveolar count, etc.) that has been demonstrated to represent perturbations in lung morphometry in premature BPD lungs.

### Brain

The brains were extracted, post-fixed in 4% paraformaldehyde solution overnight at 4°C, cryoprotected in 30% sucrose, and then serially sectioned into 16-μm coronal sections. Anterior slides were collected at −0.36 mm from Bregma (anterior commissure area), and posterior sections were collected at −2.16 mm from Bregma (hippocampus area) from all the animals. One slide was prepared for each animal; each slide included two anterior sections and three posterior sections that always were collected at the same anatomical markings to allow comparisons between animals. Each anterior section was collected at a 160 μm distance from the previous one; a similar distance between sections was used for the collection of the posterior sections (Shaikh et al., 2015; Wintermark et al., 2015).

These slides were used to assess brain structure. After the hematoxylin and eosin staining using standard protocol, the brain morphology of the sections was examined with a light microscope (Leica DM4000B LED, Leica Microsystems, Wetzlar, Hessen, Germany) with a 5× objective. For each section, overlapping microphotographs were captured using a digital camera attached to the microscope (Leica DFC450C, Leica Microsystems, Wetzlar, Hessen, Germany). These pictures were then stitched together using a panoramic image stitching software (Version 2.0.3.0; Microsoft Research Image Composite Editor, 2015) to obtain pictures of the entire coronal section. Using ImageJ (Image Processing and Analysis in Java) (Schneider et al., 2012), the size of various brain structures was measured in accordance with the Rat Brain in Sterotaxic Coordinates (Paxinos and Watson, 2006) on two anterior and posterior sections by one investigator, who was blind to the treatment group subdivision; the two measurements for each section were averaged to represent each animal. The surface of the whole brain, the width of the corpus callosum (using the midline as a reference), and the cortical thickness (from the horn of the corpus callosum to the outer limit of the brain, parallel to the midline) were measured on both the anterior and posterior sections. The surface of the anterior commissure was measured on the anterior sections. The surfaces of the hippocampus were measured on the posterior sections. These structures' measurements are one among several available brain measurements (i.e. behavioral function, neurons count, apoptosis, etc.) that have been reported to be abnormal in the context of encephalopathy of prematurity.

### Retina

The left eyes were enucleated and immediately immersed in 3.5% glutaraldehyde for 3 h, prior to the removal of the cornea and lens. The eyecups thus obtained were re-immersed in 3.5% glutaraldehyde, and left overnight at 4°C in an orbital shaker. On the following day, the eyecups were washed 3×5 min in a 0.1 M phosphate buffer. Then, the eyecups were incubated in a solution of 1% osmium tetroxide for 3 h, followed by 3×5 min washes in a 0.1 M phosphate buffer and sequential immersions in 50, 80, 90, 95, and 100% ethanol and propylene oxide prior to embedding (Epon resin, Mecalab, Montreal, QC, Canada). The embedded eyes were sectioned into 1 µm-thick sections along the superior-inferior axis at the level of the optic nerve head, collected on glass slides, and stained with 0.1% Toluidine Blue (Jung et al., 2015).

Images were taken with a microscope (Leica DM4000B LED) equipped with a digital camera (Leica DFC450C, Leica Microsystems, Wetzlar, Hessen, Germany) combined with a 40× objective. The thicknesses of the total retina and of each retinal layer were measured as per standard convention at 1000 µm from the optic nerve head in the inferior retina using AxioVision^®^ software (Version 4.9.1.0; Carl Zeiss Microscopy GmbH, Jena, Germany). For retinal reconstruction, retinal segments of 75 µm in width – taken every 310 μm along the entire length of the superior and inferior retinas – were assembled side by side (Adobe Photoshop, Adobe Systems Inc. San Jose, CA, USA) to yield a pan-retinal view. The thicknesses were then plotted against eccentricity to obtain the spider graphs, with the center of the graphs representing the optic nerve head, the left side of the graphs representing the superior retina, and the right side of the graphs representing the inferior retina (Jung et al., 2015). Retinal thickness is one among several available retinal measurements (i.e. electroretinogram, angiogenesis, etc.) that have been reported to be abnormal in the context of retinopathy of prematurity.

### Data analysis

Two-tailed Mann–Whitney *U*-tests were used to assess the differences in the lung, brain, and retinal structures between the control group and the BPD group. Using Spearman's rank correlations, we also explored the relationship between the lung, brain and retinal structures, i.e. the association between the mean linear intercept (representing the lung alveolar structure) and the whole-brain surface measured on the posterior sections (representing the brain structure); between the mean linear intercept and the average total retinal thickness (representing retinal structure); and between the whole-brain surface and the total retinal thickness. A *P* value <0.05 was considered as statistically significant. All statistical analyses were performed using GraphPad Prism (GraphPad Software Inc., San Diego, CA, USA).
